# Risk factors and protective measures for healthcare worker infection during highly infectious viral respiratory epidemics: A systematic review and meta-analysis

**DOI:** 10.1017/ice.2021.18

**Published:** 2021-01-25

**Authors:** Chenchen Tian, Olivia Lovrics, Alon Vaisman, Ki Jinn Chin, George Tomlinson, Yung Lee, Marina Englesakis, Matteo Parotto, Mandeep Singh

**Affiliations:** 1 Faculty of Medicine, University of Toronto, Toronto, Ontario, Canada; 2 Faculty of Medicine, McMaster University, Hamilton, Ontario, Canada; 3 Infection Prevention and Control, University Health Network, Toronto, Ontario, Canada; 4 Department of Anesthesia and Pain Management, University Health Network, Toronto, Ontario, Canada; 5 Department of Medicine, University Health Network, Toronto, Canada; 6 Division of General Surgery, McMaster University, Hamilton, Ontario, Canada; 7 Library and Information Services, University Health Network, Toronto, Ontario, Canada; 8 Department of Anesthesia and Pain Management, Women’s College Hospital, Toronto, Ontario, Canada

## Abstract

**Objective:**

To investigate risk factors for healthcare worker (HCW) infection in viral respiratory pandemics: severe acute respiratory coronavirus virus 2 (SARS-CoV-2), Middle East respiratory syndrome (MERS), SARS CoV-1, influenza A H1N1, influenza H5N1. To improve understanding of HCW risk management amid the COVID-19 pandemic.

**Design:**

Systematic review and meta-analysis.

**Methods:**

We searched MEDLINE, EMBASE, CINAHL, and Cochrane CENTRAL databases from conception until July 2020 for studies comparing infected HCWs (cases) and noninfected HCWs (controls) and risk factors for infection. Outcomes included HCW types, infection prevention practices, and medical procedures. Pooled effect estimates with pathogen-specific stratified meta-analysis and inverse variance meta-regression analysis were completed. We used the GRADE framework to rate certainty of evidence. (PROSPERO no. CRD42020176232, 6 April 2020.)

**Results:**

In total, 54 comparative studies were included (n = 191,004 HCWs). Compared to nonfrontline HCWs, frontline HCWs were at increased infection risk (OR, 1.66; 95% CI, 1.24–2.22), and the risk was greater for HCWs involved in endotracheal intubations (risk difference, 35.2%; 95% CI, 21.4–47.9). Use of gloves, gown, surgical mask, N95 respirator, face protection, and infection training were each strongly protective against infection. Meta-regression showed reduced infection risk in frontline HCWs working in facilities with infection designated wards (OR, −1.04; 95% CI, −1.53 to −0.33, *P* = .004) and performing aerosol-generating medical procedures in designated centers (OR, −1.30; 95% CI, −2.52 to −0.08; *P* = .037).

**Conclusions:**

During highly infectious respiratory pandemics, widely available protective measures such as use of gloves, gowns, and face masks are strongly protective against infection and should be instituted, preferably in dedicated settings, to protect frontline HCW during waves of respiratory virus pandemics.

The profound impact of the novel coronavirus (SARS-Cov-2) has been driven by the ease with which human-to-human transmission occurs, contributing to the rapid propagation of coronavirus disease 2019 (COVID-19). SARS-Cov-2 can be transmitted through cough or respiratory droplets, contact with infected bodily fluids, or less commonly, from contaminated surfaces.^1,2^

Healthcare workers (HCWs) are particularly vulnerable to SARS-CoV-2 infection and other emerging, highly infectious diseases due to close contact with infected patients and contaminated materials.^3^ Previous coronaviruses, such as severe acute respiratory syndrome coronavirus (SARS) and Middle East respiratory syndrome coronavirus (MERS), have demonstrated extensive transmission in healthcare settings even though they are relatively inefficient in transmission within the general community.^4,5^ As of July 14, 2020, data from Italy estimated that healthcare providers managing patients with COVID-19 account for 12% of cases.^6^ Factors believed to contribute to the rapid spread among healthcare workers include suboptimal infection control practices, performance of aerosol-generating medical procedures, and failure to continue adequate mask use in break rooms.^7–9^ The prevalence of infected HCWs also differs by hospital units, being highest in medical intensive care units and emergency departments.^10^

The preservation of health and wellness in HCWs is paramount because of their role in caring for critically ill patients as well as the need to prevent outbreaks in healthcare facilities.^11^ Currently, understanding of COVID-19 infection rates in HCWs and the risk factors predisposing to infection in pandemic settings is limited, and infection control guidelines across international organizations are inconsistent.^12^ Prior systematic reviews have focused on subsets of viral respiratory infections, but none have focused on risk factors for HCW infection in pandemic settings. A recent meta-analysis found protective effects of face masks, eye protection, and physical distancing in preventing virus transmission in both public and healthcare settings.^13^ Healthcare settings are unique in their challenges to financial and PPE resources, workforce availability, inherent fear, and anxiety among frontline staff, which are exacerbated during novel viral outbreaks.^14^ The current study provides a thorough review of occupational risk factors for infection in HCWs and protective measures necessary to mitigate risk in such rare and challenging times. Therefore, in this systematic review and meta-analysis, we aimed to identify risk factors for HCW infection during a WHO-classified epidemic of a highly infectious viral respiratory infection, comparable to COVID-19.

## Methods

### Search strategy and selection criteria

The study was prepared according to the Preferred Reporting Items for Systematic Review and Meta-Analysis (PRISMA) guidelines^15^ and was guided by specifications outlined in the Meta-analysis of Observational Studies (MOOSE) recommendations.^16^ The study was registered on PROSPERO (CRD42020176232) on April 6, 2020.

The search strategy was developed in consultation with a medical librarian and was conducted according to recommendations in the Cochrane Rapid Review guide.^17^ The searches were conducted in electronic databases MEDLINE, EMBASE, CINAHL, and Cochrane CENTRAL from database conception until July 6, 2020 (Appendix 1 online). We excluded case reports, case series, editorials, narrative reviews, consensus opinions, news articles, and letters to the editor. Searches were restricted to articles written in English and studies involving human subjects only.

Titles and abstracts were screened to identify potentially eligible studies, which subsequently underwent full-text review for study inclusion using predetermined inclusion and exclusion criteria. Literature screening and eligibility assessment was performed independently by 2 reviewers (C.T., O.L.) at all stages. Reasons for exclusion were documented at each stage. Data extraction was conducted independently by 2 authors (C.T., O.L.) using a standardized data extraction form. Opinions from senior authors were solicited to resolve any conflicts.

Studies were included if the study population was comprised of HCWs in a healthcare setting with pandemic respiratory disease with a similar outbreak and transmission dynamics (droplet size) to COVID-19, including MERS, SARS, H1N1, and H5N1. Studies describing nonrespiratory infectious diseases, infectious diseases not defined by the World Health Organization (WHO) as epidemic or pandemic (eg, seasonal influenza), and diseases occurring in nonhealthcare settings were excluded. HCWs were defined as all staff in a healthcare facility involved in the provision of care to patients, not only those directly providing patient care.^18^ Only comparative studies with a valid infected HCW (cases) group and a noninfected HCW (control) group were included. Therefore, studies that reported the prevalence of risk factors (described below) in both case and control groups were eligible for inclusion. We included observational studies (eg, cross-sectional, cohort, or case-control studies) and experimental studies (eg, randomized control trials [RCTs]).

### Outcomes of interest

We sought to answer 3 knowledge questions: (1) Which types of HCWs and which medical departments are at increased risk of infection? (2) Which infection prevention and control practices are associated with protective effects for infection in HCWs? (3) Which exposures or procedures are associated with infection in HCWs? We collected data related to occupational risk factors that addressed these questions using 4 outcomes (categorical variables) in the case (infected HCWs) and control (non-infected HCWs) groups. (1) We collected data related to HCW occupation type as described previously.^18^ (2) We collected data related to work department (eg, ward, emergency [ER], intensive care unit [ICU]). Frontline HCW were defined as those with high occurrence of patient face-to-face contact, including ER staff, ICU staff, and HCW who responded affirmatively to having exposure with patients. We sought to determine whether the health facility was a designated treatment center or was unidentified as a designated center. (3) We collected data related to the following infection prevention and control practices (IPAC): personal protective equipment (PPE) use (eg, surgical mask, N95 respirator or equivalent, gowns, full-body protection, eye and face protection, gloves, proper donning and doffing techniques), hand hygiene, IPAC training, vaccination status, pharmaco-prophylaxis. (4) We collected data related to exposure and procedural risks, that is, exposures to infected patients and colleagues, contaminated materials, participation in intubation or other aerosol-generating medical procedures (AGMPs).^19^

### Data analysis

All statistical analyses and the meta-analysis were performed on STATA version 15.1 software (StataCorp, College, TX)^20^ and Comprehensive Meta-Analysis version 3 software (Englewood, NJ).^21^ We performed meta-analyses using a DerSimonian and Laird random-effects model for continuous and dichotomous outcomes, wherever applicable. Pooled effect estimates were obtained by calculating the odds ratios (ORs) for dichotomous outcomes along with their respective 95% and 99% confidence intervals (CIs). A subgroup analysis was conducted for each infectious agent.

Inverse variance weighted meta-regression analysis was performed to investigate the association between study characteristics and relevant outcomes. We included categorical variables (eg, virus type, designated centre, IPAC training, and ICU status) in the meta-regression models, wherever applicable. The R^2^ statistic was used to measure the proportion of the variability in the outcome measure explained by the statistical model.

The quality of nonrandomized studies was assessed using the Newcastle-Ottawa scale (NOS) adapted to each study’s design.^22–24^ Sensitivity analyses were conducted excluding studies with higher risk of bias. Heterogeneity between studies was assessed qualitatively and quantitatively using the Higgins I^2^ statistic. Publication bias was assessed using Egger regression and visual inspection of funnel plots. Evidence was evaluated according to the Grading of Recommendations Assessment, Development, and Evaluation (GRADE) framework.^25^

## Results

**Fig. 1.**
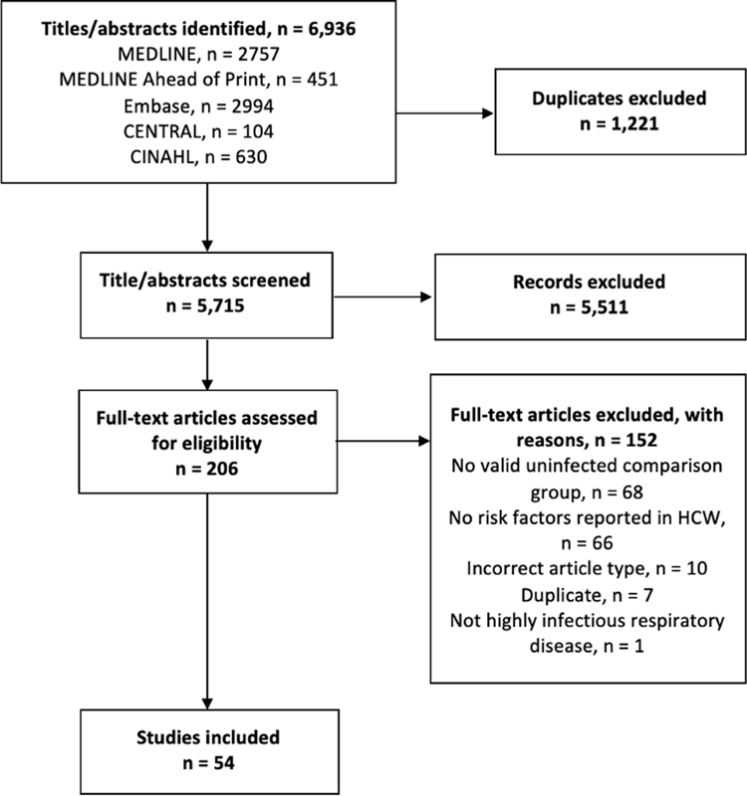
Preferred Reporting Items for Systematic Review and Meta-Analysis (PRISMA) reporting of systematic reviews and meta-analysis flow diagram outlining the search strategy results from initial search to included studies. PRISMA indicates preferred reporting items for systematic reviews and meta-analyses.

After the removal of duplicated search results, 6,936 articles underwent title and abstract screening. Of these, 204 full-text articles were assessed for eligibility for inclusion. Overall, 54 studies were included for analysis (Fig. 1).

The included studies represented a total population of 191,004 healthcare workers and 7,375 cases of confirmed infection by the pathogen under study. All included studies were comparative and observational in nature, including 28 retrospective cohort studies, 10 case-control studies, 11 prospective cohort studies, and 5 cross-sectional studies, and the studies were conducted across 5 continents among 20 countries (Table 1). The infectious agents evaluated included COVID-19 (17 studies, n = 152,019),^26–42^ H1N1 (18 studies, n = 26,349),^43–60^ SARS (15 studies, n = 6,360),^61–75^ MERS (3 studies, n = 5,750),^76–78^ and H5N1 (1 study, n = 526).^79^ No eligible RCTs were identified. The vast majority of studies (49 of 54; 90%) used WHO-defined criteria for confirmation of cases (Table 1).

**Table 1.** Study Characteristics

First Author, Year	Country	Virus Causing Disease	Study Design	Sample Size	Cases	Controls	Case Definition (WHO)	Newcastle Ottawa Scale
Bai, 2020^41^	China	COVID-19	Retrospective cohort	118	12	106	Confirmed	★★★★★
Barrett, 2020^31^	USA	COVID-19	Prospective cohort	546	40	506	Confirmed	★★★★
Chatterjee, 2020^32^	India	COVID-19	Case control	751	378	373	Confirmed	★★★★★★★
Chen, 2020	China	COVID-19	Retrospective cohort	105	18	87	Confirmed	★★★★★
El-Boghdadly 2020	UK	COVID-19	Prospective cohort	1,718	184	1,534	Probable	★★★★★★★★
Eyre, 2020^33^	UK	COVID-19	Prospective cohort	9,809	1,083	8,726	Confirmed	★★★★★★★
Guo, 2020^40^	China	COVID-19	Case control	72	24	48	Confirmed	★★★★★★★
Heinzerling, 2020^38^	USA	COVID-19	Retrospective cohort	37	3	34	Confirmed	★★★★
Houlihan, 2020^34^	UK	COVID-19	Prospective cohort	200	87	113	Confirmed	★★★★★
Korth, 2020^29^	Germany	COVID-19	Prospective Cross-sectional	316	5	311	Confirmed	★★★★★★★
Lai, 2020^37^	China	COVID-19	Retrospective cohort	9,648	110	9,538	Confirmed	★★★★★★★
Lahner, 2020^28^	Italy	COVID-19	Cross-sectional	2,115	58	2,057	Confirmed	★★★★★★★
Mani, 2020^42^	USA	COVID-19	Retrospective cohort	3,477	185	3,292	Confirmed	★★★★★
Ran, 2020^36^	China	COVID-19	Retrospective cohort	72	28	44	Confirmed	★★★★★
Wang Q, 2020^35^	China	COVID-19	Prospective cohort	5,442	120	5,322	Confirmed	★★★★★
Wang X, 2020^27^	China	COVID-19	Retrospective cohort	493	86	407	Confirmed	★★★★
Zheng, 2020^26^	China	COVID-19	Cross-sectional	117,100	2,457	114,643	Confirmed	★★★★★★★
Balkhy, 2010^43^	Saudi Arabi	H1N1	Prospective cohort	9,780	526	9,254	Confirmed	★★★★★★★
Bandaranayake, 2010^44^	New Zealand	H1N1	Retrospective cohort	532	142	390	Confirmed	★★★★★★★★
Bhadelia, 2013^53^	USA	H1N1	Retrospective cohort	352	141	211	Confirmed	★★★★★★★★
Chen, 2010	Singapore	H1N1	Prospective cohort	531	35	496	Confirmed	★★★★★★★★
Chokephaibulkit, 2012^55^	Thailand	H1N1	Retrospective cohort	256	33	223	Confirmed	★★★★★★★★
Chu, 2012^56^	Taiwan	H1N1	Retrospective cohort	4,963	51	4,912	Confirmed	★★★★★★
Hudson, 2013^52^	New Zealand	H1N1	Retrospective cohort	1,027	224	803	Confirmed	★★★★★★★★
Jaeger, 2011^58^	USA	H1N1	Retrospective cohort	63	9	54	Confirmed	★★★★★★★
Jefferies, 2011^59^	New Zealand	H1N1	Retrospective cohort	548	96	452	Confirmed	★★★★★★★★★
Kuster, 2013^60^	Canada	H1N1	Prospective cohort	563	13	550	Confirmed	★★★★★★★★
Lobo, 2013^45^	Brazil	H1N1	Case control	274	52	222	Confirmed	★★★★★★
Marshall, 2011^46^	Australia	H1N1	Prospective cohort	231	46	185	Confirmed	★★★★★★★
Nukui, 2012^47^	Japan	H1N1	Cross-sectional	438	146	292	Confirmed	★★★★★★★★
Raymond, 2012^48^	New Zealand	H1N1	Retrospective cohort	559	103	456	Confirmed	★★★★★
Sandoval, 2016^49^	Chile	H1N1	Retrospective cohort	117	34	83	Confirmed	★★★★★★★★
Toyokawa, 2011^50^	Japan	H1N1	Cross-sectional	268	14	254	Confirmed	★★★★★
Zhang, 2012^51^	China	H1N1	Case control	255	51	204	Confirmed	★★★★★★★★
Bridges, 2000^79^	China	H5N1	Retrospective cohort	526	10	516	Confirmed	★★★★★★★
Alraddadi, 2016	Saudi Arabia	MERS	Retrospective cohort	283	20	263	Confirmed	★★★★★★★★
Hastings, 2016^76^	Saudi Arabia	MERS	Retrospective cohort	4,730	16	4,714	Confirmed	★★★★★★★
Kim, 2016^78^	South Korea	MERS	Retrospective cohort	737	2	735	Confirmed	★★★★★
Caputo, 2006^61^	Canada	SARS	Retrospective cohort	33	3	30	Probable	★★★★★
Chen MIC, 2006^82^	Taiwan	SARS	Retrospective cohort	647	20	627	Probable	★★★★★★★
Chen W-Q, 2009^68^	China	SARS	Retrospective cohort	758	91	667	Confirmed	★★★★★★★★
Ho KY, 2004^83^	Singapore	SARS	Prospective cohort	303	8	295	Confirmed	★★★★★★★★
Lau, 2004^69^	Hong Kong	SARS	Case control	215	72	143	Confirmed	★★★★★★★
Liu, 2009^70^	China	SARS	Case control	477	51	426	Probable	★★★★★
Loeb, 2004^71^	Canada	SARS	Retrospective cohort	43	8	35	Probable	★★
Nishiura, 2005^72^	Vietnam	SARS	Case control	115	25	90	Confirmed	★★★★★★★★
Nishiyama, 2008^73^	Vietnam	SARS	Prospective cohort	146	59	87	Confirmed	★★★★★★★
Pei, 2006^67^	China	SARS	Case control	443	147	296	Confirmed	★★★★★★★★
Raboud, 2010^74^	Canada	SARS	Retrospective Cohort	624	26	598	Confirmed	★★★★★★★
Reynolds, 2006^75^	Vietnam	SARS	Case control	193	36	157	Confirmed	★★★
Teleman, 2004^63^	Singapore	SARS	Case control	86	36	50	Confirmed	★★★★★★
Wang F-D, 2007^64^	Taiwan	SARS	Retrospective cohort	2,197	9	2,188	Confirmed	★★★★★
Wilder-Smith, 2005^65^	Singapore	SARS	Retrospective cohort	80	45	35	Confirmed	★★★★★★★★

Note. SARS, severe acute respiratory syndrome; MERS, Middle East respiratory syndrome coronavirus; WHO, World Health Organization. Higher number of stars indicates lower risk of bias. WHO case definition in Appendix 6 (online).

Study quality ranged from poor (n = 27), to fair (n = 2), to good (n = 25) (Appendix 2 online).^80^ To adjust for study quality, sensitivity analyses including only studies with low risk of bias (NOS ≥ 7) did not yield any significant change in effect estimates for outcomes. Evidence of publication bias from visual inspection of funnel plots and the Egger test was not strongly indicative (Table 2; Appendix 3 online).

**Table 2.** Grading of Recommendations, Assessment, Development, and Evaluation (GRADE) of Meta-Analyzed Outcomes by 3 Knowledge Questions

Certainty Assessment							Summary of Findings			
No. of Studies (Total Participants)	Risk of Bias	Inconsistency	Indirectness	Imprecision	Other Considerations^†^	Overall Certainty of Evidence	Anticipated Absolute Risk (ie, Chance of Viral Infection)		Risk Difference, % (95% CI)	
							Control Risk, % (95% CI)	Intervention Risk, % (95% CI)		Anticipated Effects
**A) Knowledge Question #1: Which types of HCW are at increased risk of infection?**										
Frontline vs. non-frontline HCW										
32 (31,308)	Not serious^a^	Not serious^b^	Not serious^e^	Not serious	None	⊕⊕ 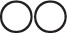 LOW	7.6	12.0 (9.3–15.4)	4.4 (1.7–7.8)	Frontline HCW may be at considerable increased risk of infection compared to non-frontline HCW.
Physicians (reference group) vs. nurses										
29 (131,794)	Not serious^a^	Not serious^b^	Not serious^e^	Not serious	None	⊕⊕ 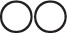 LOW	3.1	2.9 (2.4–3.5)	−0.2 (−0.7 to 0.4)	There may be little to no difference in rate of infection between physicians and nurses.
**B) Knowledge Question #2: Which infection prevention and control practice are associated with protective effects for infection in HCW?**										
Gloves										
16 (4,498)	Not serious^a^	Not serious^b^	Not serious^e^	Not serious	Strong association^g^	⊕⊕⊕ 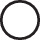 MODERATE	25.7	14.3 (9.7–20.6)	−11.5 (−16.0 to −5.1)	The use of gloves probably results in a large reduction of infection risk.
Gown										
8 (3,048)	Not serious^a^	Not serious^b^	Not serious^e^	Not serious	Strong association^g^	⊕⊕⊕ 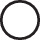 MODERATE	30.6	16.9 (9.9–16.0)	−13.7 (−20.7 to −3.3)	Gown use probably result in a large reduction of infection risk.
Surgical mask										
12 (1,960)	Not serious^a^	Not serious^bc^	Not serious^e^	Not serious	Strong association^g^	⊕⊕⊕ 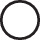 MODERATE	20.6	8.8 (4.9–14.6)	−11.9 (−15.7 to −6.0)	Surgical mask use probably results in a large reduction in infection risk.
N95 mask										
15 (9,178)	Not serious^a^	Not serious^b^	Not serious^e^	Not serious	Strong association^g^; publication bias^h^	⊕⊕⊕ 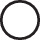 MODERATE	6.6	2.2 (1.3–3.5)	−4.4 (−5.2 to −3.0)	N95 use probably results in a large reduction of infection.
Face protection										
11 (5,116)	Not serious^a^	Not serious^b^	Not serious^e^	Not serious	Strong association^g^; publication bias^h^	⊕⊕⊕ 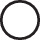 MODERATE	19.9	9.2 (6.3–13.3)	−10.6 (−13.6 to −6.5)	Wearing goggles or face shields probably results in a large reduction of infection.
Hand hygiene										
13 (3,499)	Not serious^a^	Not serious^d^	Not serious^e^	Not serious	Publication bias^h^	⊕⊕ 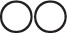 LOW	14.6	8.5 (5.5–13.0)	−6.1 (−9.1 to −1.6)	Hand hygiene may result in considerable reduction in infection risk.
Infection control and prevention training										
6 (2,589)	Not serious^a^	Not serious^b^	Not serious^e^	Not serious	Strong association^g^	⊕⊕⊕ 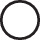 MODERATE	24.4	7.2 (4.3–12.0)	−17.1 (−20.1 to −12.4)	Infection control training probably results in a large reduction in infection risk.
H1N1 vaccine (during H1N1 pandemic)^i^										
3 (1,527)	Not serious^a^	Not serious^c^	Not serious^e^	Not serious	Strong association^g^	⊕⊕⊕ 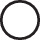 MODERATE	3.6	0.4 (0.2–0.8)	−3.2 (−3.5 to −2.8)	Receiving H1N1 vaccine probably reduces rate of H1N1 infection during an outbreak.
**C) Knowledge Question #3: What is the association of AGMPs with infection in HCW?**										
Participation in intubation procedure										
8 (3,208)	Not serious^a^	Not serious^b^	Not serious^e^	Not serious	Strong association^g^	⊕⊕⊕ 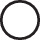 MODERATE	22.1	57.3 (43.5–70.1)	35.2 (21.4–47.9)	Involvement in intubation procedures probably causes large increases in risk of infection.
Participation in aerosol generating medical procedures, including intubation										
19 (6,897)	Not serious^a^	Not serious^b^	Not serious^e^	Not serious	Strong association^g^	⊕⊕⊕ 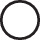 MODERATE	22.7	41.5 (31.0–52.9)	18.8 (8.3–30.2)	Performance of aerosol generating medical procedures probably results in a considerable increase in rate of infection.

a All studies were nonrandomized and evaluated using the Newcastle-Ottawa Scale (NOS). Most studies were at a lower risk of bias (NOS ≥7 stars). Furthermore, sensitivity analysis excluding studies with higher risk of bias did not yield any important difference in effect. Therefore, risk of bias was not downgraded.

b While there was a high I^2^ value, there was a large amount of overlapping of confidence intervals and low variation of effect estimates across studies. Thus, inconsistency was not downgraded.

c Low heterogeneity was detected with overall I^2^ <50% or some heterogeneity was explained through subgroup analysis demonstrating lower I2 value(s) <50%.

d Clinical heterogeneity associated with variable definitions of hand hygiene was probably introduced and inconsistency was downgraded.

e All studies included reported risk factors for health care workers infection of a highly infectious respiratory virus (SARS, H1N1, MERS, or H5N1) with a valid noninfected comparator group. Each disease-causing pathogen have caused epidemics with sufficient similarity in severity and transmission patterns. All outcomes (ie, infected cases) were ‘confirmed’ or ‘probable’ based on World Health Organization case definition criteria. Therefore, we did not rate down for indirectness of population, exposure, comparator, or outcomes.

g Magnitude of effect is large considering the thresholds set by GRADE (RR >2 or <0.5) with consistent evidence from at least 2 studies. Effect size assumes that the odds ratios translate into similar magnitudes of relative risk estimates.

h Although publication bias was suggested through the Egger test, visual inspection of funnel plots was largely symmetrical and thus, we did not downgrade for strongly suspected publication bias.

i No other virus-specific immunizations were identified in the literature.

^f^Downgraded 1 point because of large confidence intervals that overlaps both little to no effect, as well as appreciable benefit or appreciable harm of the intervention/exposure. This suggests that more studies with larger sample sizes are needed to calculate precise effect estimate.

**Fig. 2.**
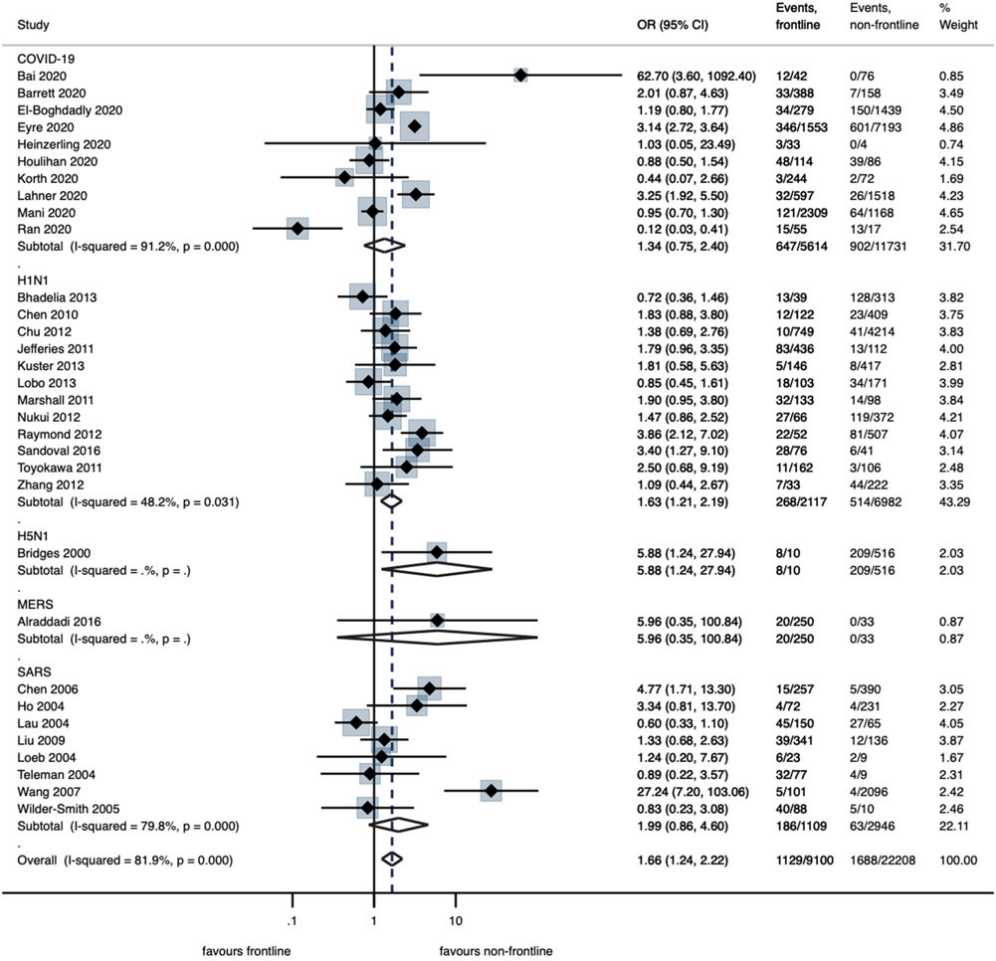
Forest plot of random effect meta-analysis of the risk of infection in frontline healthcare workers (HCWs) by virus type. Frontline HCWs were defined as those with high occurrence of patient face-to-face contact, including emergency department staff, intensive care unit staff, and HCWs who responded affirmatively to having direct exposure with patients.

Infection rates in frontline HCWs were analyzed from 32 studies^28,29,31,33,34,36,38,39,41,42,45–51,53,54,56,59,60,63–65,69–71,79,81–83^ and were significantly higher in this group of HCWs compared to nonfrontline HCWs (OR, 1.66; 95% CI, 1.24–2.22, *P* = .001; 12.0% in frontline vs 4.4% non-frontline; low certainty) (Fig. 2; Table 2). Meta-regression analysis using random effects was performed by including covariates, wherever applicable. The overall risk of infection was higher among frontline workers (2-sided *P* = .039; τ^2^ = .3435; R^2^ = 72%). Furthermore, working within a designated center versus an unidentified center was protective (OR, − 1.04, 95%CI, −1.53 to −0.33; *P* = .004) (Table 1; Appendix 5, Fig. 1 online). Our model was unable to detect statistical difference in infection risk between the various virus types (*P* = .566). Similarly, there was low certainty that the difference in overall infection rates between physicians and nurses was not statistically significant (Table 2; Appendix 4, Fig. 1 online).^26,28,30–32,34–37,41,43–45,49–51,54–57,67,70,73–76,81,82,84^

**Fig. 3.**
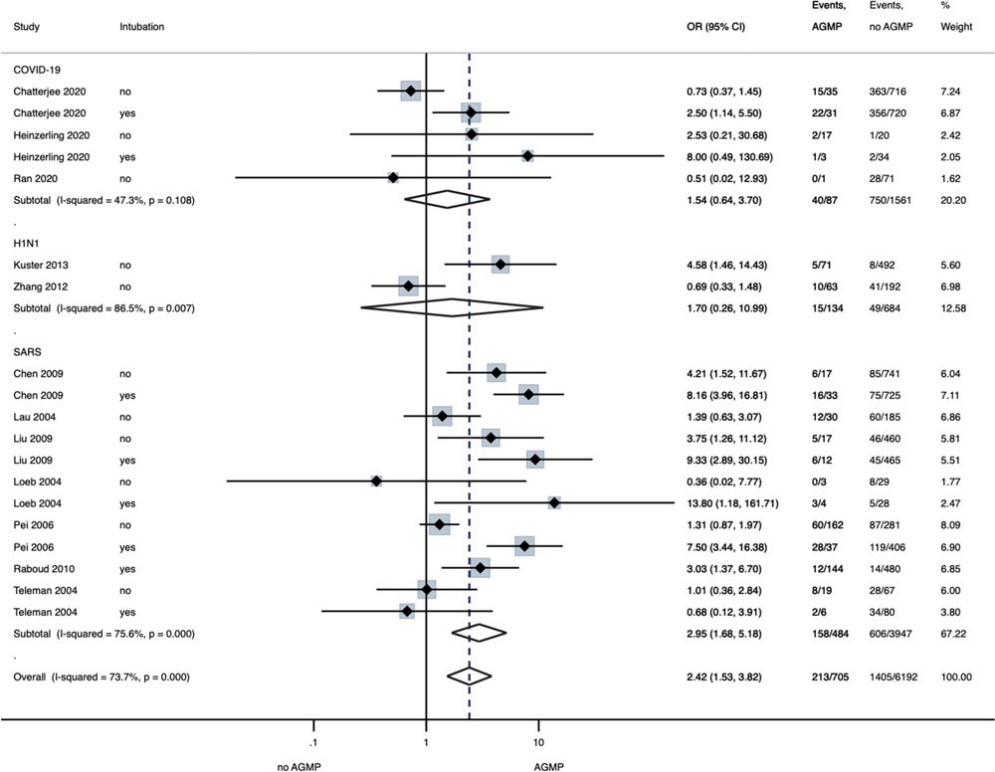
Forest plot of random effect meta-analysis of the association of aerosol-generating medical procedures (AGMPs) on infection in HCWs by virus type. AGMPs include endotracheal intubation, chest compressions, and other airway manipulations.

**Fig. 4.**
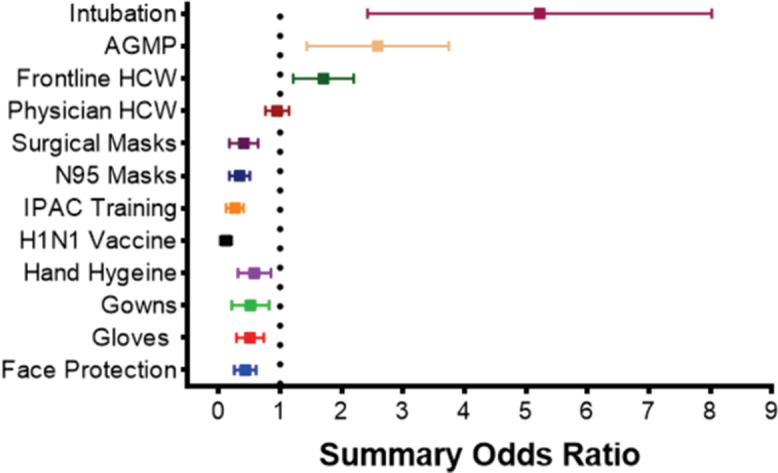
Forest plot of all the summary odds ratios for meta-analysed risk factors. *Represents the overall odds ratios for meta-analysed risk factors on healthcare worker infection during all included viral respiratory pandemics. Comparator groups: intubation versus no intubation; AGMP versus no AGMP; frontline HCW versus non-frontline HCW; physician versus nurse; surgical mask versus no surgical mask; N95 mask versus no N95 mask; IPAC training versus no IPAC training; hand hygiene versus no hand hygiene; gowns versus no gowns; gloves versus no gloves; face protection versus no face protection.

Compared to control (ie, no use of corresponding PPE item), use of gloves (16 studies)^31,32,38,46,50,51,55,58,61,63,65,67,69,70,72,74^, gowns (8 studies)^31,32,50,63,67,69,72,74^, surgical masks (12 studies)^38,49–51,55,58,67,70–73,75^, N95 respirators (15 studies)^27,35,40,50,51,55,61,63,65,69–71,74,78,81^, and face protection (11 studies)^32,50,60,61,67–70,74,77,78^ were associated with large reductions in the risk of infection (moderate certainty; Table 2; Appendix 4, Figs. 2–6 online). The definition of N95 respirator use varied greatly across studies. The 2 studies with the strongest effects for use of N95 respirators both investigated COVID-19, but they did not clearly define the setting in which this occurred.^27,35^ Furthermore, most studies of N95 respirators did not provide detail on the comparison group (eg, surgical mask, no mask) and had varying definitions for the use of N95 respirators, such as use all of the time,^40^ always while in an infected patient’s room,^74,85^ or during intubation.^61^

Across 13 studies,^36,40,49–51,55,60,63,65,68,69,72,74^ compared to controls (no hand hygiene), hand hygiene following exposure to patients showed an overall significant protective effect (OR, 0.54; 95% CI, 0.34–0.87; *P* = .012) (low certainty; Table 2; Appendix 4, Fig. 7 online). IPAC training (6 studies^40,67–70,74^) was associated with a large reduction in infection risk (OR, 0.24; 95 CI%, 0.14–0.42; *P* < .001) with an overall risk reduction of 17.1% (95% CI, 12.4%–20.1%; moderate certainty; Table 2; Appendix 4, Fig. 8 online). Compared to no H1N1 vaccine, H1N1 vaccine was strongly protective during the H1N1 pandemic (OR, 0.10; 95% CI, 0.04–0.22; *P* < .001) (moderate certainty; Appendix 4, Fig. 9 online).^51,57,60^

Compared to control (no involvement in intubation procedures), involvement in intubation (8 studies^32,38,63,67,68,70,71,74^) was associated with a significant increase in infection risk (OR, 4.72; 95% CI, 2.71–8.24; *P* < .001) (57.3% in intubation vs 22.1% in no intubation; moderate certainty) (Table 2; Appendix 4, Fig. 10 online). Across 19 studies,^32,36,38,51,60,63,67–71,74^ a composite measure of AGMPs was associated with significant increased risk of infection (OR, 2.42; 95% CI, 1.53–3.82; *P* < .001) (41.5% in AGMPs vs 22.7% in no AGMPs; moderate certainty; Fig. 3; Table 2). On subgroup analysis, significantly increased odds of infection were only seen with SARS (OR, 2.95; 95% CI, 1.68–5.18; *P* < .001) and not for COVID-19 or H1N1 (Fig. 3). Meta-regression analysis, including covariates of designated status (designated center vs unidentified center), IPAC measures (implemented vs unimplemented or undefined), AGMP type (intubation vs other AGMPs), ICU versus non-ICU, and virus type was performed (τ^2^ = 0.2428; I^2^ = 73%; R^2^ = 0.61) (Appendix 5, Table 2 online). The rate of infection associated with performing AGMPs was a significantly lower in designated facilities compared than in those not identified as such (OR, −1.30; 95% CI, −2.52 to −0.08; *P* = .037). A higher rate of infection was associated with intubation compared to other AGMPs (OR, 1.04; 95% CI, 0.30–1.77; *P* = .006) (Appendix 5, Fig. 3 online).

Summary odds ratios for meta-analyzed risk factors are reported in Figure 4. To emphasize the meta-analysed effect estimates of risk factors with greater robustness, additional meta-analysis with 99% confidence intervals were conducted. In this analysis, risk factors with effect estimates that persisted toward significant effect included frontline HCW, gloves, surgical masks, N95 masks, face protection, IPAC training, H1N vaccination, intubation, and participation in AGMPs (Appendix 6 online).

## Discussion

This systematic review and meta-regression analysis provides a comprehensive summary of occupational risk factors for HCW infection during viral respiratory pandemics. Our findings suggest that compared to nonfrontline HCWs, frontline HCWs are at significantly increased risk of infection during an outbreak (low certainty). Use of gloves, gowns, surgical masks, N95 respirators, and face protection, as well as receiving IPAC training were each associated with large reductions in infection (moderate certainty). Compared to other AGMPs, endotracheal intubation of patients with coronaviruses SARS-CoV-1 and SARS-CoV-2 was associated with a very large increase in the HCW infection rate (moderate certainty). Meta-regression analysis revealed that the availability of isolation wards was protective from infection among frontline HCWs and those performing AGMPs.

The safety of HCWs is paramount for many reasons, including the facilitation of continuous patient care, prevention of virus infection for themselves and also spread to other patients, as well as an ethical duty to protect those who put themselves on the frontline to treat others. The results of our review demonstrate the efficacy of well-known measures, such as PPE adherence and IPAC training, against viral respiratory pathogens that have stood as pillars of infection prevention and control in healthcare settings. The delivery of adequate IPAC training also poses its own barriers, including constantly changing guidelines, poor communication and enforcement of guidelines, and increased workload and fatigue in HCWs, which may be heightened during a pandemic lasting many months.^86^ Thus, despite the novelty of SARS-CoV-2, it is likely that interventions long-practiced in acute-care sites across the globe are adequate to protect frontline staff against the virus.^87^

Our findings regarding the protective effects of PPE use and increased transmission risk associated with AGMPs are generally consistent with results from previous reviews in the HCW population.^13,88–91^ A recent rapid review reported that in healthcare settings, risk for infection with SARS-CoV-1 was likely decreased with mask use versus no mask use and possibly decreased with N95 versus surgical mask use, with uncertain applicability to SARS-CoV-2 due to lack of direct evidence, This finding is generally consistent with our report relating to mask effectiveness and SARS-CoV-2.^92^ Of the 3 studies reporting a significant increase in risk for involvement in AGMPs, these procedures included endotracheal intubation and nebulization therapy with inconsistent reports of PPE use during the procedure.^60,68,70^ Critically, none of these 3 studies addressed whether proper PPE was worn by personnel during these procedures, including use of N95 respirators. Based on these and other findings, national guidelines therefore universally recommend N95 respirators during AGMPs performed on patients with COVID-19.^93–96^

The strengths of this study are that it identified a multitude of different factors relating to infection risk during previous respiratory viral epidemics representative worldwide through stringent methodology of data synthesis. Nearly all included studies met the WHO criteria for confirmed positive cases for each respective disease, ensuring the accuracy of cases and controls (Appendix 7 online). Our review highlights respiratory viruses with transmission profiles and reproductive numbers comparable to SARS-CoV-2, thereby increasing the generalizability of our findings and their applicability to the ongoing pandemic, distinct from previous reviews.^90,97,98^ Finally, we used the GRADE approach to facilitate transparent recommendations and interpretations of the data.^25^

Although stringent methods were adhered to, limitations were inherent in the current review. First, randomized trials were lacking due to the inherent ethical risk of restricting protective measures during an emerging epidemic. Most studies were of retrospectively design, potentially leading to selection and measurement biases and failure to match for potential confounding variables such as age, sex, and baseline comorbidities.^91^ We also observed also heterogeneity introduced in the meta-analysis of many unique viral pathogens, each with different epidemiological profiles. Furthermore, the differences in global impact of the various pathogens (8,098 worldwide SARS-CoV-1 cases versus 56 million worldwide SARS-CoV-2 cases and increasing) introduced heterogeneity in meta-analyzed risk factors, potentially reducing the certainty of evidence for certain findings.^99,100^ We conducted a pathogen-specific stratified meta-analysis to address these differences. However, few individual patient factors were reported (eg, ethnicity, sociodemographic factors, and comorbidity status) that likely influence HCW susceptibility to infection.^101^ Emerging literature suggests that black, Asian, and minority ethnic individuals are at increased risk of SARS-CoV-2 infection, with worse clinical outcomes.^102^ Heterogeneity was observed in classifying the various risk factors. Few studies have explored the role of HCW-to-HCW transmission of pathogens, which has been associated with an increased risk of SARS-CoV-2 transmission in HCW without adherence to medical mask use in break rooms and during meals.^9^ Moreover, data on compliance with hand hygiene or proper donning and doffing technique and staff surveillance strategies were limited, and both of these factors have been shown to be critical in reducing the infection risk.^90,103,104^ These limitations were addressed by conducting meta-regression, controlling for virus type, and various covariates, and thereby adjusted estimates provide a conservative assessment of the risk to HCWs. The protective effects of each individual PPE item may be confounded by the reality that PPE is usually worn in bundles (eg, mask with face shield, gloves, and gown) and therefore may not reflect the true effect estimates of each PPE item, and these protective effects may be additive in when adhering to PPE bundles. Lastly, restriction of articles to the English language, to produce a timely review, may have excluded potentially relevant studies.

Amid the evolving COVID-19 pandemic, rapidly released research has attempted to answer many questions regarding the safety of HCWs caring for patients with COVID-19. Our review has shown that some key questions remain to be answered, including efforts to report detailed data for ethnicity, sociodemographic factors and comorbidity status, and direct head-to-head comparison of N95 respirators and surgical masks in the routine care of patients with COVID-19, a topic which has yet to be directly addressed by current evidence.^98,101,105^

In conclusion, this systematic review and meta-analysis synthesizes the current evidence for the risk of infection among HCWs in a viral respiratory outbreak and draws attention to useful protective strategies while caring for patients, especially for frontline HCWs performing risk-prone exposures. IPAC measures should be instituted, preferably in dedicated settings, to protect frontline HCWs during current and future waves of respiratory virus pandemics.

## Supplementary material

For supplementary material accompanying this paper visit http://dx.doi.org/10.1017/ice.2021.18. click here to view supplementary material
