# Supplementary material for: Risk factors and protective measures for healthcare worker infection during highly infectious viral respiratory epidemics: A systematic review and meta-analysis
Source: Infect Control Hosp Epidemiol. 2021 Jan 25:1–12. doi: 10.1017/ice.2021.18 (PMC8564050; doi:10.1017/ice.2021.18)
Supplement: Supplementary file 1 [file S0899823X21000180sup001.docx]

Supplementary material

Appendix 2: Newcastle-Ottawa scale

| **Study** | **Virus causing disease** | **Selection** | **Comparability** | **Outcome/**  **Exposure** | **Overall Rating** | **Quality** |
| --- | --- | --- | --- | --- | --- | --- |
| Alraddadi, 2016 | MERS | ★★★ | ★★ | ★★★ | ★★★★★★★★ | Good |
| Bai, 2020 | COVID-19 | ★★ | - | ★★★ | ★★★★★ | Poor |
| Balkhy, 2010 | H1N1 | ★★★★ | - | ★★★ | ★★★★★★★ | Poor |
| Bandaranaya, 2010 | H1N1 | ★★★ | ★★ | ★★★ | ★★★★★★★★ | Good |
| Barrett, 2020 | COVID-19 | ★★ | - | ★★ | ★★★★ | Poor |
| Bhadelia, 2013 | H1N1 | ★★★★ | ★★ | ★★ | ★★★★★★★★ | Good |
| Bridges, 2000 | H5N1 | ★★★ | ★ | ★★★ | ★★★★★★★ | Good |
| Caputo, 2006 | SARS | ★★ | - | ★★★ | ★★★★★ | Poor |
| Chatterjee, 2020 | COVID-19 | ★★★ | ★★ | ★★ | ★★★★★★★ | Good |
| Chen, 2006 | SARS | ★★★ | ★★ | ★★ | ★★★★★★★ | Good |
| Chen, 2009 | SARS | ★★★★ | ★★ | ★★ | ★★★★★★★★ | Good |
| Chen, 2010 | H1N1 | ★★★ | ★★ | ★★★ | ★★★★★★★★ | Good |
| Chen, 2020 | COVID-19 | ★ | ★ | ★★★ | ★★★★★ | Poor |
| Chokephaibulkit, 2012 | H1N1 | ★★★ | ★★ | ★★★ | ★★★★★★★★ | Good |
| Chu, 2012 | H1N1 | ★★★ | - | ★★★ | ★★★★★★ | Poor |
| Costa, 2012 | H1N1 | ★★★ | - | ★★★ | ★★★★★★ | Poor |
| El-Boghdadly 2020 | COVID-19 | ★★★ | ★★ | ★★★ | ★★★★★★★★ | Good |
| Eyre 2020 | COVID-19 | ★★★ | ★★ | ★★ | ★★★★★★★ | Good |
| Guo, 2020 | COVID-19 | ★★★★ | - | ★★★ | ★★★★★★★ | Poor |
| Hastings, 2016 | MERS | ★★★★ | - | ★★★ | ★★★★★★★ | Poor |
| Heinzerling, 2020 | COVID-19 | ★★ | - | ★★ | ★★★★ | Poor |
| Ho, 2004 | SARS | ★★★ | ★★ | ★★★ | ★★★★★★★★ | Good |
| Houlihan 2020 | COVID-19 | ★★★ | - | ★★ | ★★★★★ | Poor |
| Hudson, 2013 | H1N1 | ★★★ | ★★ | ★★★ | ★★★★★★★★ | Good |
| Jaeger, 2011 | H1N1 | ★★★★ | - | ★★★ | ★★★★★★★ | Poor |
| Jefferies, 2011 | H1N1 | ★★★★ | ★★ | ★★★ | ★★★★★★★★★ | Good |
| Kim, 2016 | MERS | ★★★★ | - | ★★ | ★★★★★★ | Poor |
| Korth, 2020 | COVID-19 | ★★★ | ★ | ★★★ | ★★★★★★★ | Good |
| Kuster, 2013 | H1N1 | ★★★ | ★★ | ★★★ | ★★★★★★★★ | Good |
| Lahner, 2020 | COVID-19 | ★★★ | ★★ | ★★ | ★★★★★★★ | Good |
| Lai, 2020 | COVID-19 | ★★★★ | - | ★★★ | ★★★★★★★ | Poor |
| Lau, 2004 | SARS | ★★★★ | ★★ | ★ | ★★★★★★★ | Poor |
| Liu, 2009 | SARS | ★★★ | ★ | ★ | ★★★★★ | Poor |
| Lobo, 2013 | H1N1 | ★★★ | ★★ | ★ | ★★★★★★ | Poor |
| Loeb, 2004 | SARS | ★★ | - | - | ★★ | Poor |
| Mani, 2020 | COVID-19 | ★★ | ★ | ★★ | ★★★★★ | Fair |
| Marshall, 2011 | H1N1 | ★★★ | ★★ | ★★ | ★★★★★★★ | Good |
| Nishiura, 2005 | SARS | ★★★ | ★★ | ★★★ | ★★★★★★★★ | Good |
| Nishiyama, 2008 | SARS | ★★★ | ★★ | ★★ | ★★★★★★★ | Good |
| Nukui, 2012 | H1N1 | ★★★ | ★★ | ★★★ | ★★★★★★★★ | Good |
| Pei, 2006 | SARS | ★★★ | ★★ | ★★★ | ★★★★★★★★ | Good |
| Raboud, 2010 | SARS | ★★ | ★★ | ★★★ | ★★★★★★★ | Fair |
| Ran, 2020 | COVID-19 | ★★ | - | ★★★ | ★★★★★ | Poor |
| Raymond, 2012 | H1N1 | ★★★ | - | ★★ | ★★★★★ | Poor |
| Reynolds, 2006 | SARS | ★★ | - | ★ | ★★★ | Poor |
| Sandoval, 2016 | H1N1 | ★★★ | ★★ | ★★★ | ★★★★★★★★ | Good |
| Teleman, 2004 | SARS | ★★★ | ★★ | ★ | ★★★★★★ | Poor |
| Toyokawa, 2011 | H1N1 | ★★ | - | ★★★ | ★★★★★ | Poor |
| Wang, 2007 | SARS | ★★ | - | ★★★ | ★★★★★ | Poor |
| Wang Q, 2020 | COVID-19 | ★★★ | - | ★★ | ★★★★★ | Poor |
| Wang X, 2020 | COVID-19 | ★★ | - | ★★ | ★★★★ | Poor |
| Wilder-Smith 2005 | SARS | ★★★ | ★★ | ★★★ | ★★★★★★★★ | Good |
| Zhang, 2012 | H1N1 | ★★★★ | ★★ | ★★ | ★★★★★★★★ | Good |
| Zheng, 2020 | COVID-19 | ★★★★ | - | ★★★ | ★★★★★★★ | Poor |
|  |  |  |  |  |  |  |

**Table 1.** Newcastle-Ottawa scoring system for non-randomized studies. Cohort, case-control, or cross-sectional iterations of the scoring system were used accordingly as per individual study design. Dash (-) indicates no stars awarded, and therefore high risk of bias. Higher number of stars indicates lower risk of bias. Overall quality rated according to Agency for Healthcare Research and Quality (AHRQ) guidelines available: <https://effectivehealthcare.ahrq.gov/>.

Appendix 3: Funnel plots for publication bias using 95% confidence limits and Egger’s test (p-value)

| Frontline vs. non-frontline  p=0.131   | Physician (reference) vs nurse;  p=0.528   | \| Gloves  p=0.977   \| Physician vs nurse \| Gloves vs no gloves \| \| --- \| --- \| --- \| \|  \| Hand hygiene vs no hand hygiene when contact with infected patient \| Received infection control and prevention training \| |
| --- | --- | --- | --- | --- | --- | --- | --- | --- |
| Gown;  p=0.548   | Surgical mask;  p=0.429   | N95 respirator;  p=0.013   |
| Face/eye protection;  p=0.028   | Hand hygiene vs no hand hygiene; p=0.029   | Received infection control and prevention training; p=0.561   |
| H1N1 vaccine vs no H1N1 vaccine during H1N1 pandemic; p=0.263   | Participation in intubation procedure;  p=0.922   | Participation in AGMP, including intubation  p=0.400   |

**Appendix 4: Forest plots for other risk factors using random-effects meta-analysis by virus type**

| Figure 1. Physician (reference) vs nurse.   | Figure 2. Gloves vs no gloves   |
| --- | --- |
| Figure 3. Gown vs no gown.   | Figure 4. Surgical mask vs no surgical mask.   |

| Figure 5. N95 vs no N95 respirator.   | Figure 6. Face protection vs no face protection   |
| --- | --- |

| Figure 7. Hand hygiene vs no hand hygiene.   | Figure 8. Infection prevention training vs no training   |
| --- | --- |

| Figure 9. H1N1 vaccine vs no H1N1 vaccine during H1N1 pandemic.   | Figure 10. Intubation vs no intubation.   |
| --- | --- |

Appendix 5: Meta-regression analysis

**Table 1. Meta‐regression to evaluate the effect of study characteristics on rate of frontline HCW infection.**

| **Covariate/Model** | **Estimate** | **Lower 95% CI** | **Lower 95% CI** | **p-value** | τ^2^ | **I^2^** | **R^2^** |
| --- | --- | --- | --- | --- | --- | --- | --- |
| Model |  |  |  | 0.0391 | 0.3435 | 72% | 0.09 |
| Intercept | 1.78 | -1.27 | 4.84 | 0.252 |  |  |  |
| Designated center  (Ref: not designated) | -1.04 | -1.75 | -0.33 | 0.004 |  |  |  |
| Virus type  (Ref: MERS): |  |  |  |  |  |  |  |
| COVID-19 | -1.40 | -4.50 | 1.71 | 0.379 |  |  |  |
| H1N1 | -1.01 | -4.09 | 2.08 | 0.522 |  |  |  |
| H5N1 | -0.01 | -3.63 | 3.60 | 0.994 |  |  |  |
| SARS | -1.06 | -4.16 | 2.05 | 0.505 |  |  |  |

Included covariates: Intercept, setting (designated center vs. undefined), virus type

τ^2^: total between-study variance (intercept only)

I^2^: describes the percentage variation between studies due to heterogeneity rather than chance

R^2^: estimates the amount of heterogeneity that is accounted for by the covariates included in the meta‐regression model

**Figure 1**. Meta‐regression to evaluate the effect of **A)** designated center status and **B)** virus type, on rate of frontline HCW infection.*

**A)** **
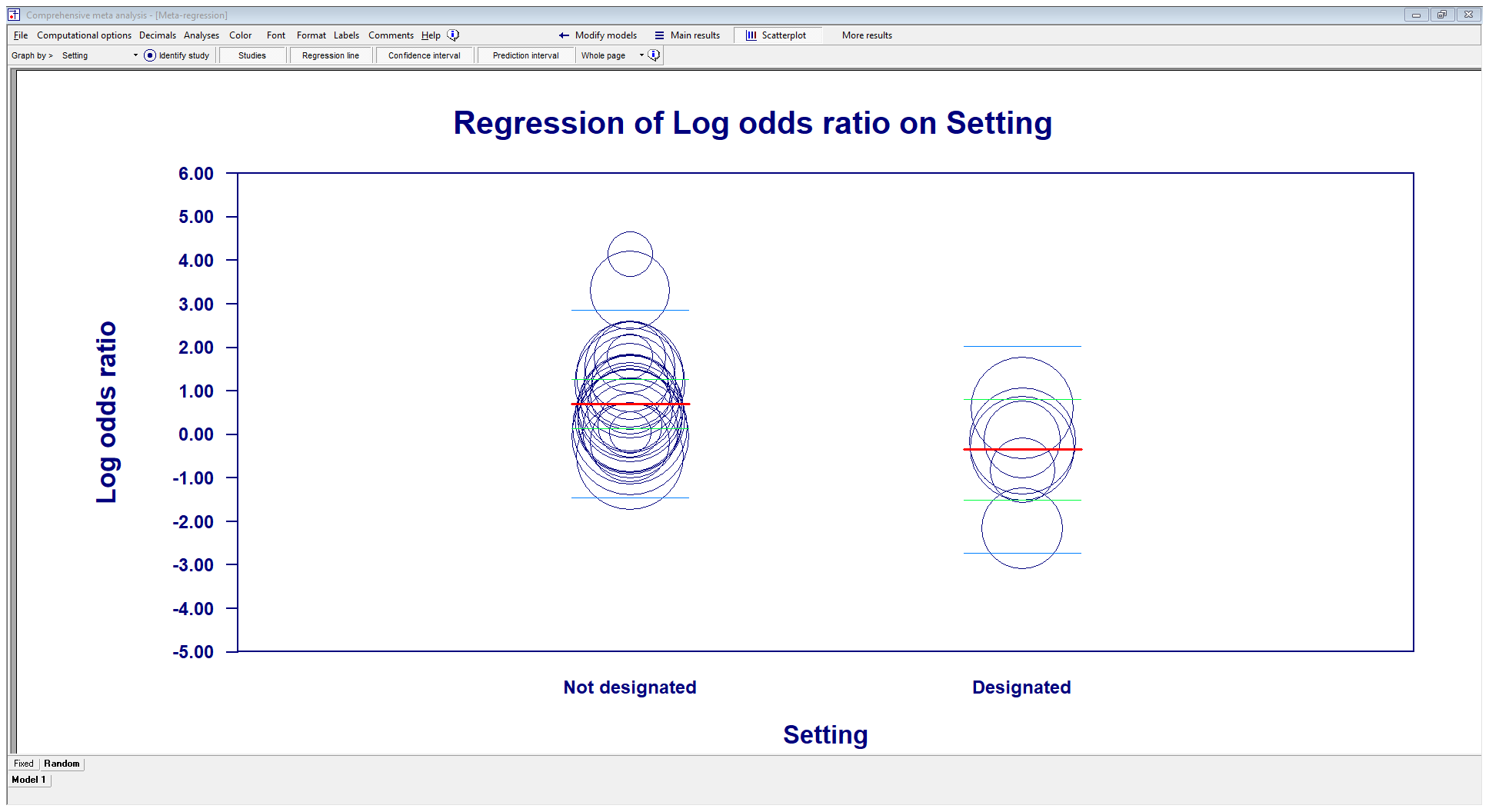
**

**B)
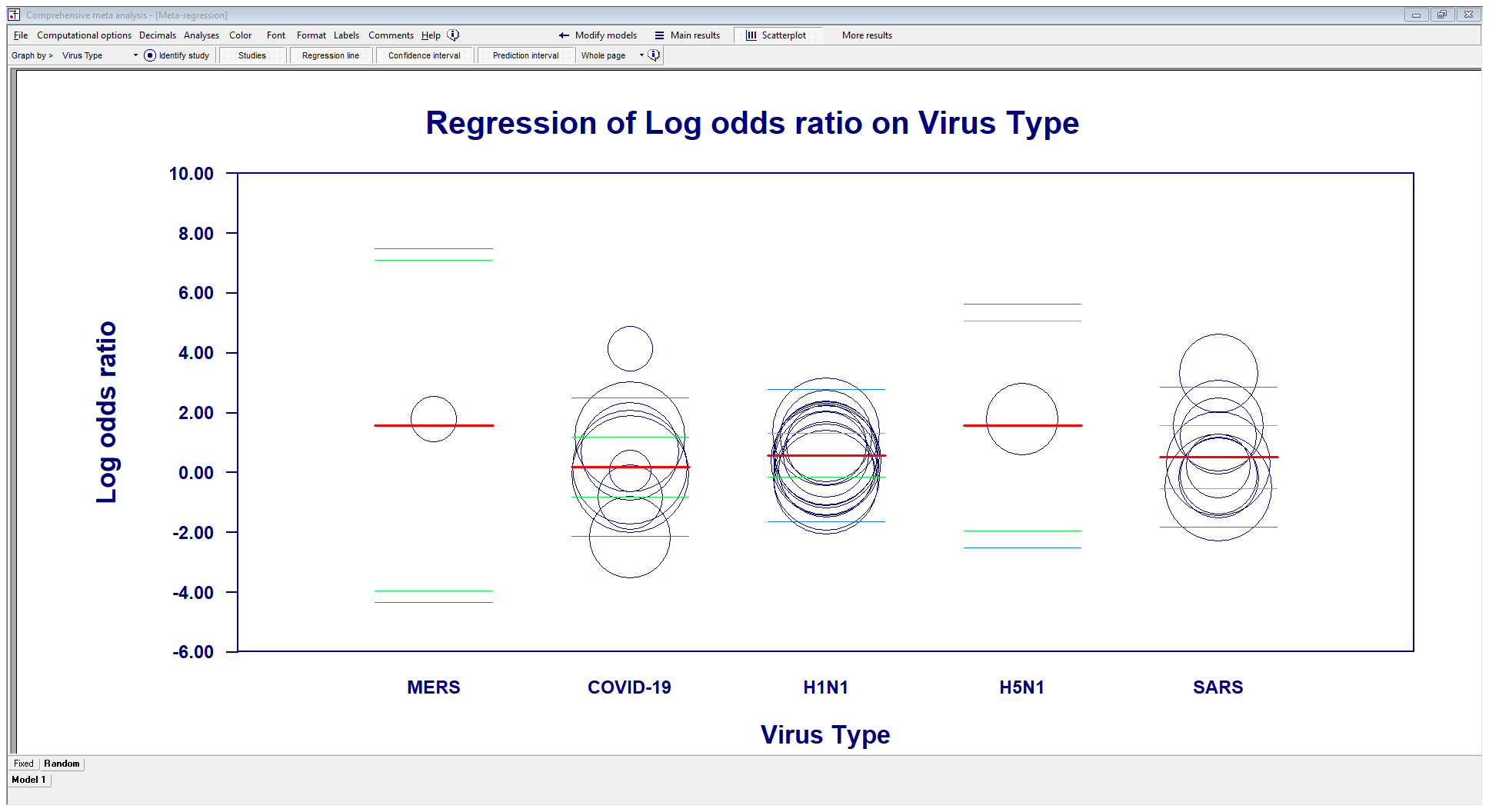
**

* The mean effect size is indicated by the regression line [bold red colour]. Where the confidence interval is an index of precision for the sampled population, the *mean effect size*for a study at any point probably falls in the confidence interval [green colour]. The prediction interval is an index of dispersion for the sampled population, the *true effect size*for a single study at any given point probably falls in the prediction interval [light blue colour].

**Table 2: Meta‐regression to evaluate the effect of study characteristics on rate of infection in HCW involved in aerosol generating medical procedures (AGMP).**

| **Covariate/Model** | **Estimate** | **Lower 95% CI** | **Lower 95% CI** | **p-value** | τ^2^ | **I^2^** | **R^2^** |
| --- | --- | --- | --- | --- | --- | --- | --- |
| Model |  |  |  | 0.005 | 0.2428 | 73% | 0.61 |
| Intercept | 0.688 | -0.563 | 1.94 | 0.281 |  |  |  |
| Designated center  (Ref: Not designated) | -1.30 | -2.52 | -0.080 | 0.037 |  |  |  |
| AGMP: intubation  (Ref: non-intubation AGMP) | 1.04 | 0.300 | 1.77 | 0.006 |  |  |  |
| IPAC defined (Ref: IPAC undefined) | -0.294 | -1.11 | 0.519 | 0.478 |  |  |  |
| ICU  (Ref: non-ICU) | -1.93 | -5.22 | 1.36 | 0.250 |  |  |  |
| Virus type (Ref: H5NI): |  |  |  |  |  |  |  |
| COVID-19 | -0.437 | -1.71 | 0.837 | 0.501 |  |  |  |
| SARS | 0.224 | -0.972 | 1.42 | 0.714 |  |  |  |

Included covariates: Intercept, setting (designated center vs. undefined), AGMP type (intubation versus other AGMP), infection prevention and control (IPAC) measures (adequately defined/implemented versus undefined or unimplemented), virus type

τ ^2^: total between-study variance (intercept only)

I^2^: describes the percentage variation between studies due to heterogeneity rather than chance

R^2^: estimates the amount of heterogeneity that is accounted for by the covariates included in the meta‐regression model

**Figure 2**. Summary of meta-regression of study characteristics on rate of infection in frontline HCWs.


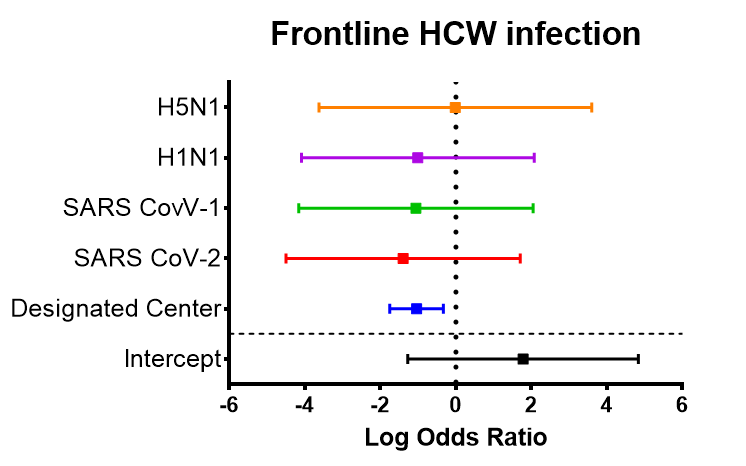


**Figure 3.** Meta‐regression to evaluate the effect of study characteristics on rate of infection in HCW involved in aerosol generating medical procedures (AGMP)*: **A)** designated vs. non-designated centers; **B)** intubation vs. other AGMP; **C)** defined IPAC measures vs. undefined; **D)** virus type; **E)** ICU vs. non-ICU

**A)** **
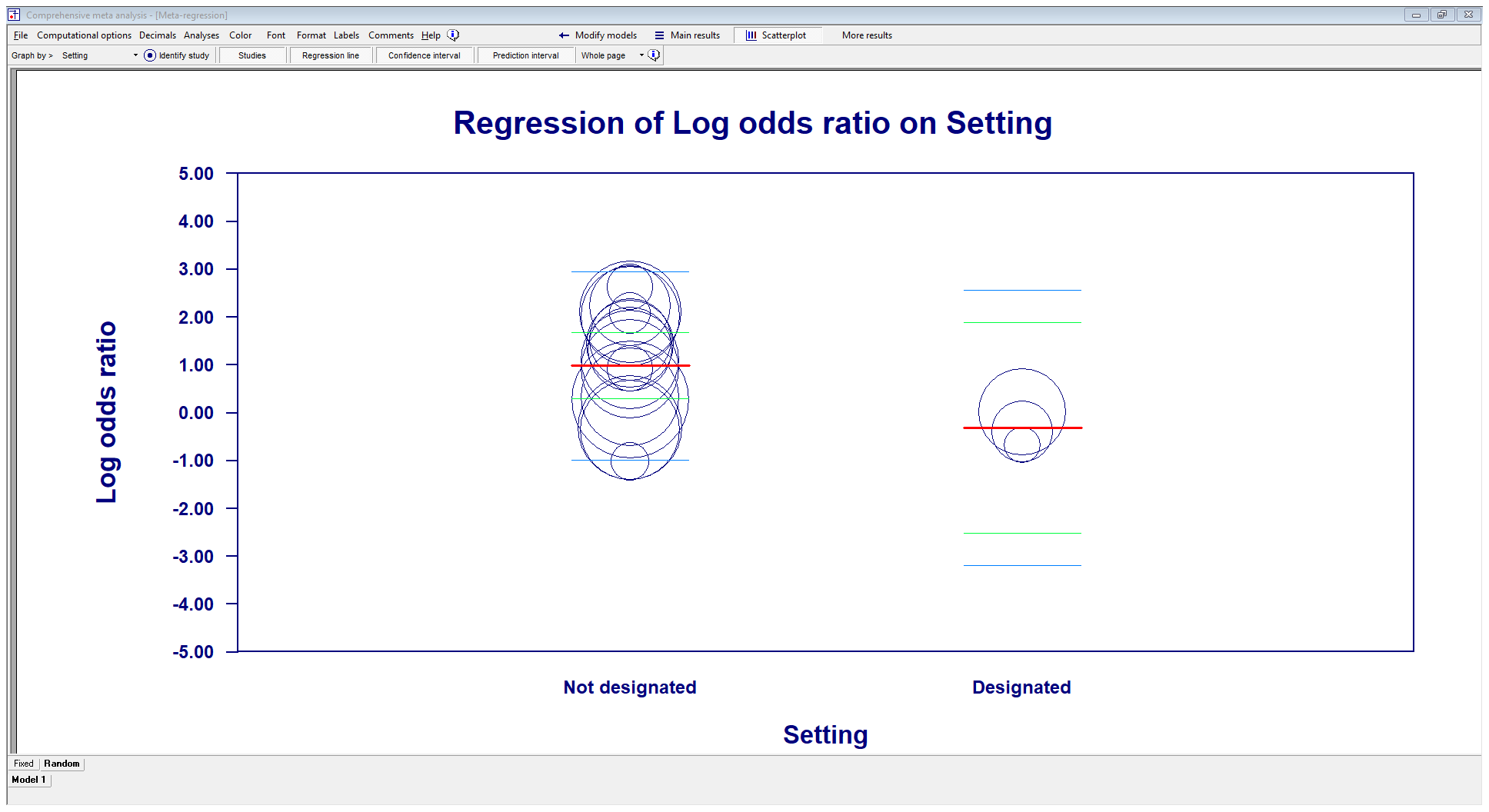
**

**B)** **
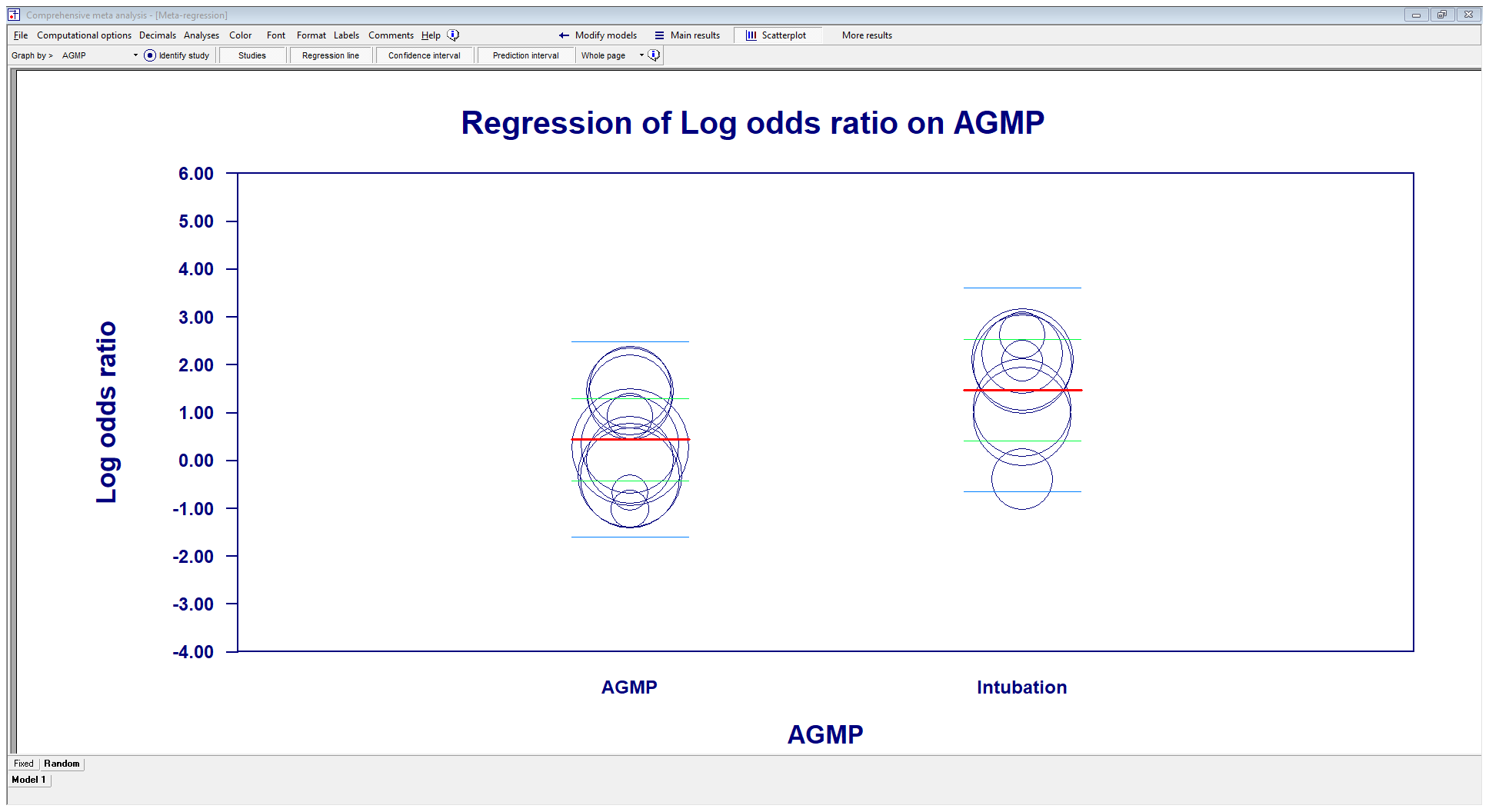
**

**C)**
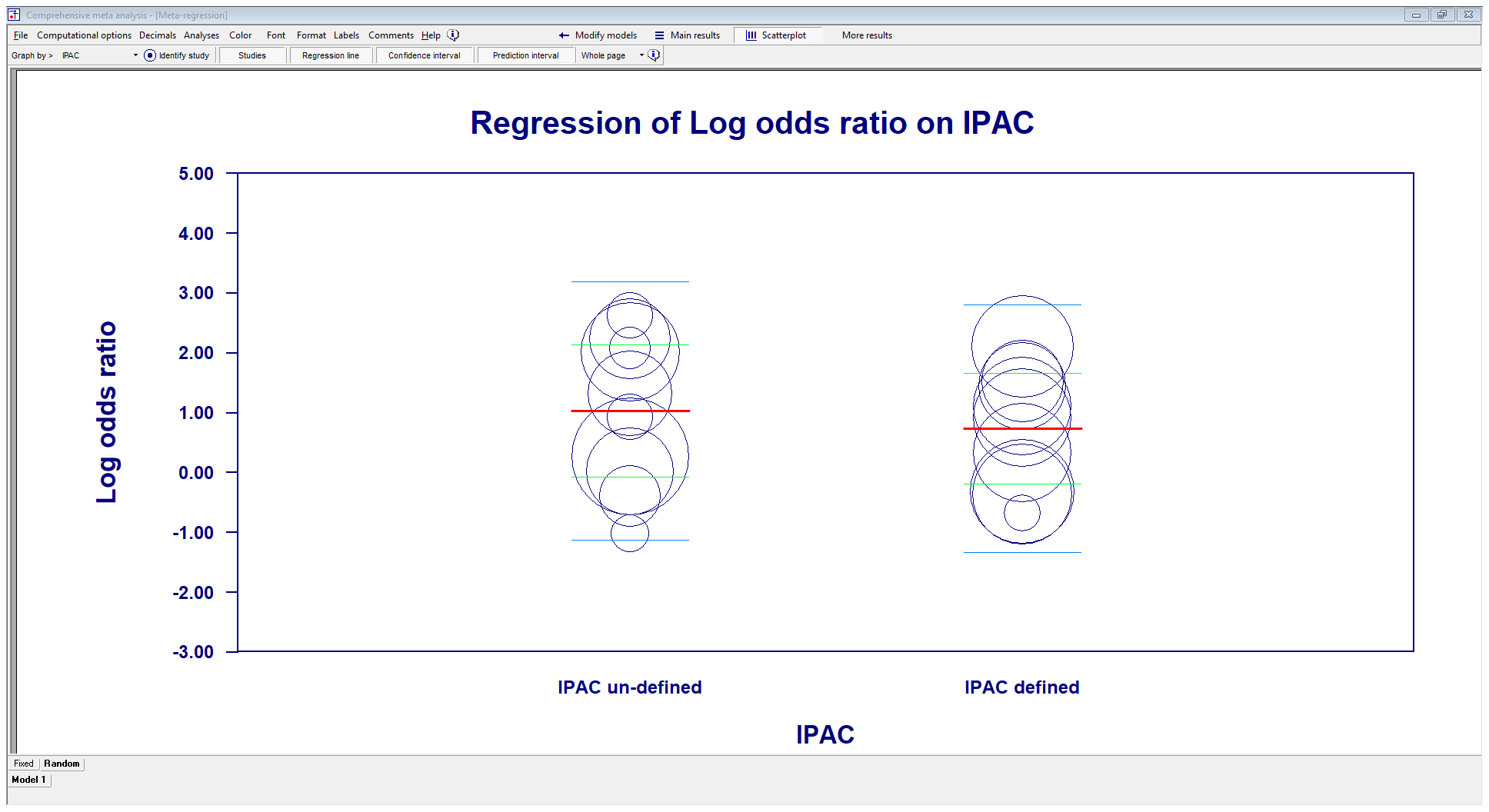


**D)** *
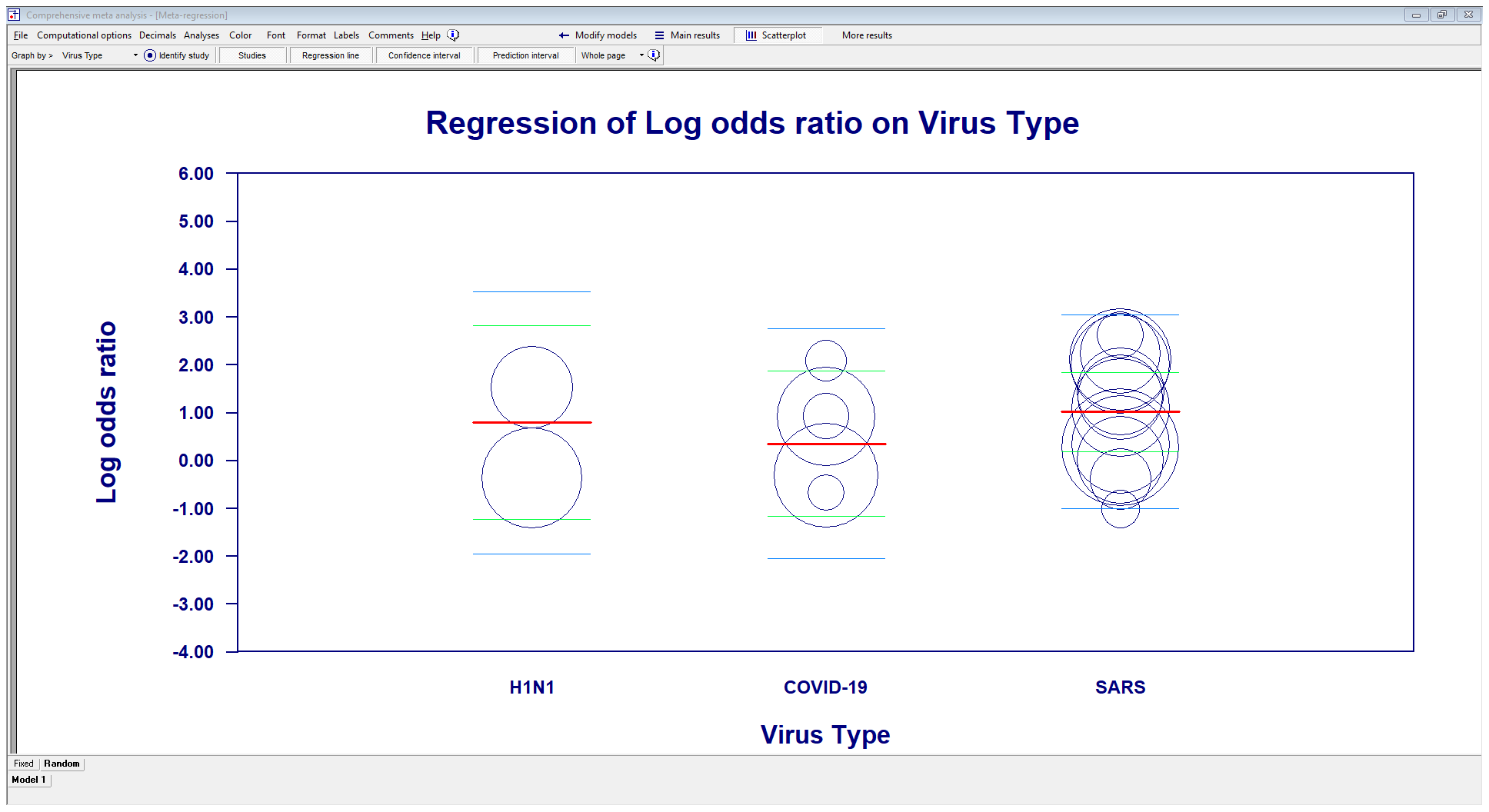
*

**E)** **
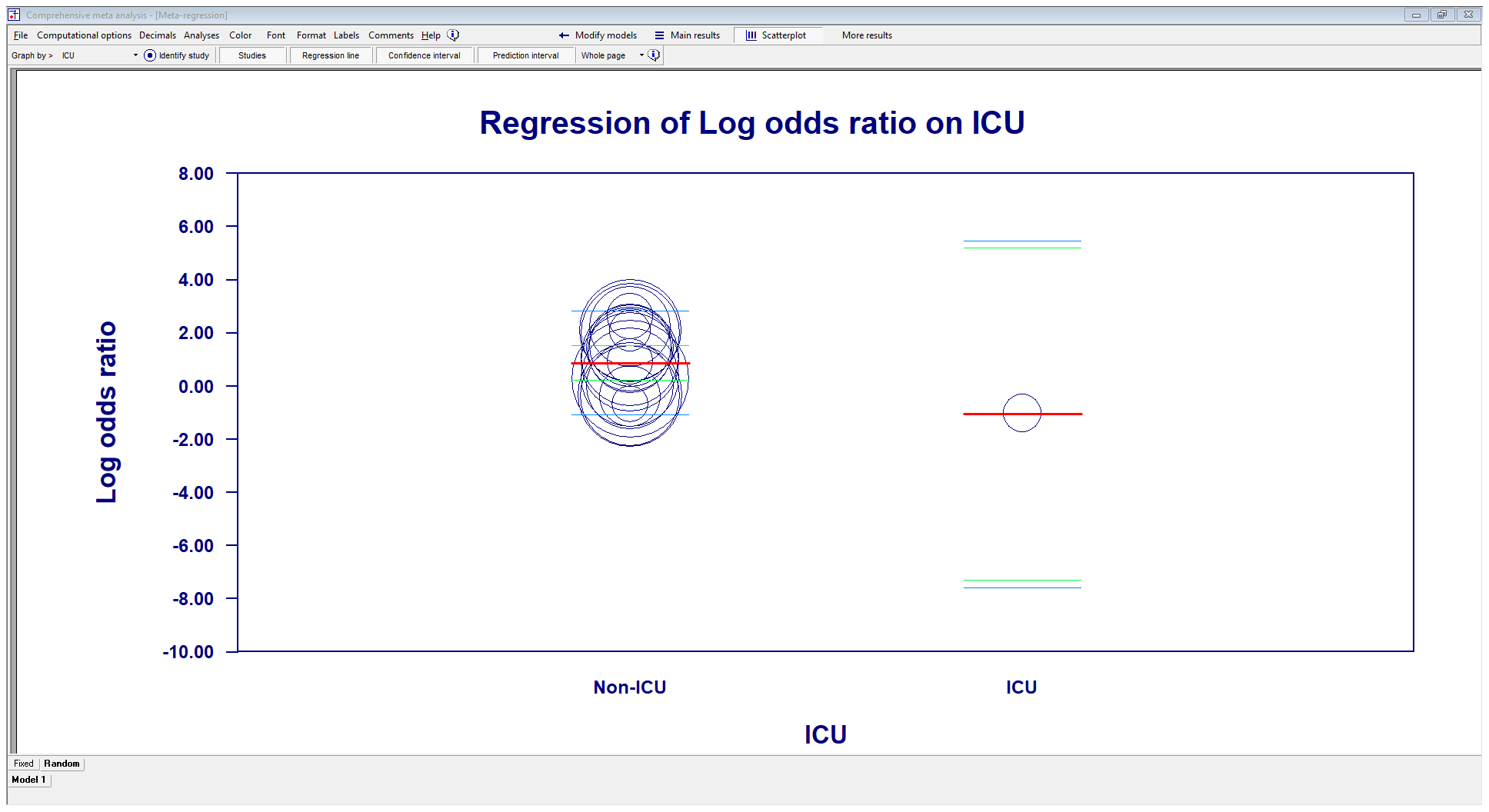
**

* The mean effect size is indicated by the regression line [bold red colour]. Where the confidence interval is an index of precision for the sampled population, the *mean effect size*for a study at any point probably falls in the confidence interval [green colour]. The prediction interval is an index of dispersion for the sampled population, the *true effect size*for a single study at any given point probably falls in the prediction interval [light blue colour].

**Figure 4**. Summary of meta-regression of study characteristics on rate of HCW infection during AGMPs.


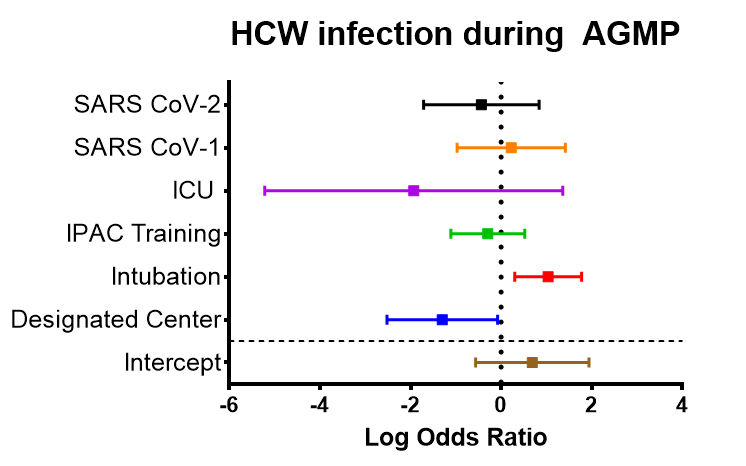


IPAC, infection control and prevention; ICU, intensive care unit; AGMP, aerosol generating medical procedure

Appendix 6:

| **Risk factor** | **Pooled OR** | **95% CI** | **99% CI** | **p-value (overall effect)** | **I^2^ (%)** |
| --- | --- | --- | --- | --- | --- |
| Frontline HCW vs non-frontline HCW | 1.66 | 1.24-2.22 | 1.13–2.43 | 0.001 | 82 |
| Physicians (reference group) vs. nurses | 0.94 | 0.77-1.12 | 0.72-1.23 | 0.563 | 69 |
| Gloves | 0.48 | 0.31-0.75 | 0.27-0.86 | 0.001 | 75 |
| Gown | 0.46 | 0.25-0.85 | 0.21-1.03 | 0.014 | 82 |
| Surgical Mask | 0.37 | 0.20-0.66 | 0.17-0.79 | 0.001 | 72 |
| N95 mask | 0.32 | 0.19-0.52 | 0.17-0.61 | <0.001 | 52 |
| Face protection | 0.41 | 0.27-0.62 | 0.24-0.70 | <0.001 | 57 |
| Hand Hygiene | 0.54 | 0.34-0.87 | 0.29-1.02 | 0.012 | 59 |
| Infection control and prevention training | 0.24 | 0.14-0.42 | 0.12-0.50 | <0.001 | 75 |
| H1N1 vaccine (during H1N1 pandemic) | 0.10 | 0.04-0.22 | 0.03-0.28 | <0.001 | 33 |
| Participation in intubation procedure | 4.72 | 2.71-8.24 | 2.27-9.81 | <0.001 | 51 |
| Participation in aerosol generating medical procedures, including intubation | 2.42 | 1.53-3.82 | 1.32-4.41 | <0.001 | 74 |
|  |  |  |  |  |  |

**Supplementary Table.** Meta–analysed odds ratios of the risk factors for HCW infection.

HCW, healthcare worker

Appendix 7: World Health Organization Diagnostic Criteria for epidemic viruses

**World Health Organization Diagnostic Criteria for SARS:** A suspect case with radiographic evidence of infiltrates consistent with pneumonia or respiratory distress syndrome on chest xray, a suspected case of SARS that is positive for SARS coronavirus by one or more assays, and/or a suspect case with autopsy findings consistent with the pathology of RDS without an identifiable cause. (<https://www.who.int/csr/sars/casedefinition/en/>)

**World Health Organization Diagnostic Criteria for H5N1:**

#### Case definitions

**Person under investigation**

A person whom public health authorities have decided to investigate for possible H5N1 infection.

**Suspected H5N1 case**

A person presenting with unexplained acute lower respiratory illness with fever (>38 ºC ) and cough, shortness of breath or difficulty breathing.

AND

One or more of the following exposures in the 7 days prior to symptom onset:

a. Close contact (within 1 metre) with a person (e.g. caring for, speaking with, or touching) who is a suspected, probable, or confirmed H5N1 case;

b. Exposure (e.g. handling, slaughtering, defeathering, butchering, preparation for consumption) to poultry or wild birds or their remains or to environments contaminated by their faeces in an area where H5N1 infections in animals or humans have been suspected or confirmed in the last month;

c. Consumption of raw or undercooked poultry products in an area where H5N1 infections in animals or humans have been suspected or confirmed in the last month;

d. Close contact with a confirmed H5N1 infected animal other than poultry or wild birds (e.g. cat or pig);

e. Handling samples (animal or human) suspected of containing H5N1 virus in a laboratory or other setting.

**Probable H5N1 case (notify WHO)**

*Probable definition 1*:
A person meeting the criteria for a suspected case

AND

One of the following additional criteria:

a. infiltrates or evidence of an acute pneumonia on chest radiograph plus evidence of respiratory failure (hypoxemia, severe tachypnea)

OR

b. positive laboratory confirmation of an influenza A infection but insufficient laboratory evidence for H5N1 infection.

*Probable definition 2*:
A person dying of an unexplained acute respiratory illness who is considered to be epidemiologically linked by time, place, and exposure to a probable or confirmed H5N1 case.

**Confirmed H5N1 case (notify WHO)**

A person meeting the criteria for a suspected or probable case

AND

One of the following positive results conducted in a national, regional or international influenza laboratory whose H5N1 test results are [accepted by WHO as confirmatory](http://www.who.int/csr/disease/avian_influenza/guidelines/labcriteria/en/):

a. Isolation of an H5N1 virus;

b. Positive H5 PCR results from tests using two different PCR targets, e.g. primers specific for influenza A and H5 HA;

c. A fourfold or greater rise in neutralization antibody titer for H5N1 based on testing of an acute serum specimen (collected 7 days or less after symptom onset) and a convalescent serum specimen. The convalescent neutralizing antibody titer must also be 1:80 or higher;

d. A microneutralization antibody titer for H5N1 of 1:80 or greater in a single serum specimen collected at day 14 or later after symptom onset and a positive result using a different serological assay, for example, a horse red blood cell haemagglutination inhibition titer of 1:160 or greater or an H5-specific western blot positive result. <https://www.who.int/influenza/resources/documents/case_definition2006_08_29/en/#:~:text=A%20person%20whom%20public%20health,of%20breath%20or%20difficulty%20breathing.>)

**World Health Organization Diagnostic Criteria for H1N1:** An individual with laboratory‐confirmed pandemic (H1N1) 2009 virus infection by one or more of the following tests: polymerase chain reaction (PCR); viral culture;  4‐fold rise in pandemic (H1N1) 2009 virus virus‐specific neutralizing antibodies. (<https://www.who.int/csr/disease/swineflu/guidance/surveillance/WHO_case_definition_swine_flu_2009_04_29.pdf?ua=1>)

**World Health Organization Diagnostic Criteria for MERS Coronavirus: Confirmed case**

A person with laboratory confirmation of MERS-CoV infection (1) irrespective of clinical signs and symptoms.

**Probable case**

1. A febrile acute respiratory illness with clinical, radiological, or histopathological evidence of pulmonary parenchymal disease (e.g. pneumonia or Acute Respiratory Distress Syndrome) AND

   Direct epidemiologic link (2) with a laboratory-confirmed MERS-CoV case AND

   Testing for MERS-CoV is unavailable, negative on a single inadequate specimen (3) or inconclusive (4)
2. A febrile acute respiratory illness with clinical, radiological, or histopathological evidence of pulmonary parenchymal disease (e.g. pneumonia or Acute Respiratory Distress Syndrome) that cannot be explained fully by any other etiology AND

   The person resides or travelled in the Middle East, or in countries where MERS-CoV is known to be circulating in dromedary camels or where human infections have recently occurred AND

   Testing for MERS-CoV is inconclusive (4)
3. An acute febrile respiratory illness of any severity AND

   Direct epidemiologic link (2) with a confirmed MERS-CoV case AND

   Testing for MERS-CoV is inconclusive (4).

**(**<https://www.who.int/csr/disease/coronavirus_infections/case_definition/en/>)

**World Health Organization Diagnostic Criteria for COVID-19:** Suspect case A. A patient with acute respiratory illness (fever and at least one sign/symptom of respiratory disease, e.g., cough, shortness of breath), AND a history of travel to or residence in a location reporting community transmission of COVID-19 disease during the 14 days prior to symptom onset. OR B. A patient with any acute respiratory illness AND having been in contact with a confirmed or probable COVID-19 case (see definition of contact) in the last 14 days prior to symptom onset; OR C. A patient with severe acute respiratory illness (fever and at least one sign/symptom of respiratory disease, e.g., cough, shortness of breath; AND requiring hospitalization) AND in the absence of an alternative diagnosis that fully explains the clinical presentation. Probable case A. A suspect case for whom testing for the COVID-19 virus is inconclusive. a. Inconclusive being the result of the test reported by the laboratory. OR B. A suspect case for whom testing could not be performed for any reason. Confirmed case A person with laboratory confirmation of COVID-19 infection, irrespective of clinical signs and symptoms. **(**<https://www.who.int/docs/default-source/coronaviruse/situation-reports/20200321-sitrep-61-covid-19.pdf>)
